# Continuous quantification of HER2 expression by microfluidic precision immunofluorescence estimates *HER2* gene amplification in breast cancer

**DOI:** 10.1038/srep20277

**Published:** 2016-02-09

**Authors:** Diego G. Dupouy, Ata Tuna Ciftlik, Maryse Fiche, Déborah Heintze, Bettina Bisig, Laurence de Leval, Martin A. M. Gijs

**Affiliations:** 1 Laboratory of Microsystems, Ecole Polytechnique Fédérale de Lausanne, CH-1015, Switzerland; 2 Institute of Pathology, Centre Hospitalier Universitaire Vaudois and University of Lausanne, CH-1011 Lausanne, Switzerland; 3 Lunaphore Technologies SA, EPFL Innovation Park—Building C, CH-1015, Lausanne, Switzerland

## Abstract

Chromogenic immunohistochemistry (IHC) is omnipresent in cancer diagnosis, but has also been criticized for its technical limit in quantifying the level of protein expression on tissue sections, thus potentially masking clinically relevant data. Shifting from qualitative to quantitative, immunofluorescence (IF) has recently gained attention, yet the question of how precisely IF can quantify antigen expression remains unanswered, regarding in particular its technical limitations and applicability to multiple markers. Here we introduce microfluidic precision IF, which accurately quantifies the target expression level in a continuous scale based on microfluidic IF staining of standard tissue sections and low-complexity automated image analysis. We show that the level of HER2 protein expression, as continuously quantified using microfluidic precision IF in 25 breast cancer cases, including several cases with equivocal IHC result, can predict the number of *HER2* gene copies as assessed by fluorescence *in situ* hybridization (FISH). Finally, we demonstrate that the working principle of this technology is not restricted to HER2 but can be extended to other biomarkers. We anticipate that our method has the potential of providing automated, fast and high-quality quantitative *in situ* biomarker data using low-cost immunofluorescence assays, as increasingly required in the era of individually tailored cancer therapy.

With the advancement of personalized cancer medicine, precise molecular profiling of tumors is gaining significant importance in routine diagnostic pathology^1^,^2^. With the evolution towards personalized treatments tailored to the molecular features of malignant tumors, the last decade has witnessed an increasing use of molecular analysis approaches, including but not limited to *in situ* hybridization (ISH), mRNA expression profiling techniques and next generation sequencing (NGS). Immunohistochemistry (IHC), however, remains by far the most used method in the routine diagnostic evaluation of tumor tissues, with the advantages of wide availability, low cost, and preservation of the information-rich morphological context. While ELISA and Western blotting are also useful protein quantification techniques and eventually can be used for testing large amounts of cells of controlled HER2 status, they require fairly large lysed samples^3^ and therefore are less suitable for assessing protein expression levels in the morphological context of the tissue slide.

Continuous quantification of protein expression in tumor sections has long been the missing link between methods analyzing nucleic acids and conventional IHC. The majority of IHC tests currently used in clinical diagnosis cannot quantify the antigen (Ag) expression but rather perform a binary or semi-quantitative assessment as interpreted by the pathologist^4^. An example of such semi-quantitative tests is the assessment of HER2 protein expression level in breast cancer, for which the scoring can have four different levels: 0, 1+, 2+ or 3+^5^. This non-continuous assessment results in a loss of information regarding the Ag expression level^6^,^7^. Comparison studies between IHC and FISH methods for HER2 have been widely performed in clinical research^8^,^9^. Yet, if a routine method that precisely quantifies Ag expression in tissues while preserving the morphology could be established, not only would this reduce the requirement for expensive complementary gene analysis but also increase the precision of diagnosis, prognosis and the success of targeted therapies, in clinical trials and routine patient care.

In this context, as clinical pathology moves from qualitative to quantitative, immunofluorescence (IF) is gaining relevance in the research settings and laboratory-developed tests, mainly due to its increased capacity to measure the signal intensity of one or more biomarkers as compared to traditional chromogenic techniques^7^,^10^. Several image processing techniques that quantify the extent of IF signal have already been reported in the literature^11^,^12^. However, there is little or no evidence suggesting that the IF signal *per se* can be used to precisely quantify Ag expression amount on tissue sections. Indeed, due to the kinetics of Ag-antibody (Ab) binding, a 2-step IF assay does not result in a signal that is linearly proportional to the Ag expression^13^,^14^, which potentially ends up in a misleading quantification and, hence, obscures the potential of IF in providing precise biomarker data. There is therefore a need to find out how precise IF can be in continuously quantifying tissue biomarkers. IF has a high potential to quickly replace routine chromogenic stain-based diagnostic IHC, since it would use the same primary antibodies, established sample preparation techniques and, hence, involve the less significant change from current laboratory practice. Therefore if a proportional relationship between signal intensity and Ag expression could be established, this could eventually allow IF to precisely quantify the Ag expression level, and evolve as a routine diagnostic tool that establishes the missing link.

We have recently introduced a microfluidic tool, called “Microfluidic Tissue Processor” (MTP) that increased the accuracy of semi-quantitative IF biomarker scoring in histopathological tissue sections^15^. However, it just provided a better semi-quantitative scoring, and the use of this technology still did not show the quantification of the biomarker expression. Yet, this technology has a high potential to be a starting point in reaching continuous quantitative IF. Here, we introduce microfluidic precision IF, a method that precisely quantifies the target Ag expression in a continuous scale based on double staining of standard tissue sections with MTP and low-complexity automated image analysis. In the following, we selected 25 invasive breast carcinoma cases whose HER2 biomarker expression ranged across a large range of values known by routine diagnostics, as assessed by the two conventional methods used (*i.e*.: IHC and FISH), and for which several cases presented equivocal results. Using these cases, we show that, continuous quantification of HER2 protein expression by microfluidic precision immunofluorescence, one can estimate the number of copies of the coding gene, as assessed by FISH. Later, in a proof-of-concept experiment, we prove that the MTP fine-tunes immunoreactions on the tissue surface in a way that allows an IF staining to establish a linearly proportional relationship between the IF signal intensity and its Ag expression level. Therefore, we propose that the working principle is not restricted to the above cases, and in principle, can be extended to all biomarkers. We anticipate that our method has the potential to provide automated and precise continuous quantitative *in situ* biomarker data using low-cost immunofluorescence assays, as increasingly required for personalized cancer therapy.

## Results

### Continuous signal quantification of double IF staining using microfluidic precision immunofluorescence

**Figure 1:**
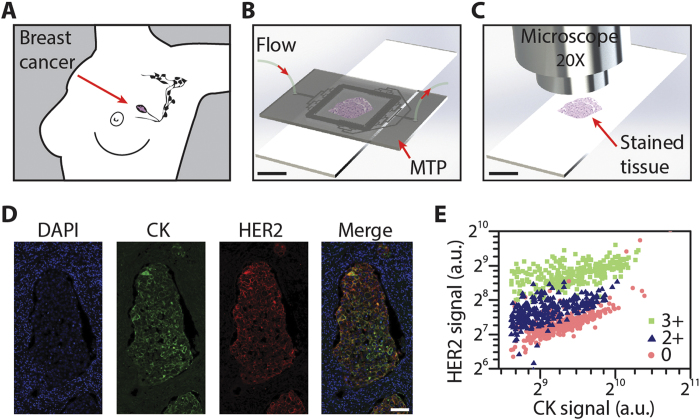
Microfluidic precision immunofluorescence. (**A**) A breast tumor is surgically resected and is to be prepared in the form of thin histological tissue slides for analysis. (**B**) A slide is clamped with the MTP for IF staining. (**C**) Subsequently the slide is coverslipped with a DAPI-containing mounting solution and imaged using a fluorescent microscope. (**D**) Mosaic images of the stained slide are acquired in 3 fluorescent channels, corresponding to the signals of DAPI, CK, and HER2, respectively, and are automatically and tile-by-tile analyzed. (**E**) 2D scatter plot showing the correlation between the averaged HER2 and CK signal per tile, for three samples with different IHC score obtained from routine analysis: 3+ (green), 2+ (blue) and 0 (red). Scale bars: 10 mm for B and C, 100 μm for D. Figures A to C were drawn by Diego Dupouy.

For this proof-of-concept study, we used the MTP to perform IF staining assays on formalin-fixed paraffin-embedded (FFPE) sections of surgically resected human invasive breast carcinoma samples (Fig. 1A), retrieved from the archives of the Institute of Pathology at the University Hospital of Lausanne (Switzerland). By clamping the MTP half-chamber with the tissue slide (Fig. 1B), a shallow flow chamber with a height of 100 μm is formed, which constitutes a critical parameter of the working principle of the device^15^, allowing fast and uniform delivery and washing of the reagents over a large surface (16 × 16 mm^2^) of tissue section. The chosen microfluidic architecture allows distributing the bioreagents uniformly within the chamber. To minimize variations in the exposure time of tissue sections to the bioreagents, the transport of the latter should rely minimally on diffusion within in-plane directions. To address this problem, we designed a distributed microfluidic channel network that permitted homogeneous flow throughout the entire chamber and assured that convection was the dominant mechanism for the in-plane bioreagent transport.

This resulted in an IF signal that was more proportional to the Ag concentration than what could be obtained by traditional IHC methods. The samples were first incubated with primary antibodies for HER2 and cytokeratin (CK), in a sequential manner^16^,^17^,^18^. CK constitutes a marker for epithelial cells and it has been widely used in carcinomas to distinguish epithelia from stroma^19^,^20^,^21^. Furthermore, CK-based normalization has proven to be a useful technique to increase accuracy in the quantification of HER2, by compensating for variations in the staining quality of the samples^22^. In a second step, two fluorescently labeled secondary antibodies were sequentially delivered into the chamber, first for HER2 and then for CK detection. Finally the slides were washed with deionized water and cover-slipped using a solution containing 4′6-diamidino-2-phenylindole (DAPI) for nuclear counterstaining. After the staining process, the slides were automatically tile-by-tile scanned to obtain a mosaic image in three fluorescent channels, corresponding to the signals of DAPI, CK and HER2, respectively (Fig. 1C,D). Each tile from the resulting mosaic images was then analyzed by running a custom-made image-processing algorithm that identified locations of CK expression and created a region of interest to limit the interrogation of the HER2 signal to epithelial areas only. Similarly, the information from the DAPI channel was used to remove the nuclei from the interrogation zones, as the HER2 and CK markers of interest are not expressed in the nuclei.

### Signal quantification data lead to distinct scatter plot signatures

As a result of the image-processing algorithm, 2D scatter plots were obtained, in which the averaged HER2 and CK signals per tile were represented as points. Figure 1E is an example of such scatter plot, in which an IHC HER2 2+ (equivocal) case is compared to a 3+ and a 0 case, the latter two being used as positive and negative controls, respectively. The data from each scatter plot were subsequently processed to provide statistical indicators of HER2 expression, which finally resulted in the ‘MTP-score’ of our analysis (see further).

### Scatter plot signatures of HER2 protein expression in 25 breast cancer cases obtained by microfluidic precision IF, and their corresponding *HER2* copy number as obtained by routine FISH

**Figure 2:**
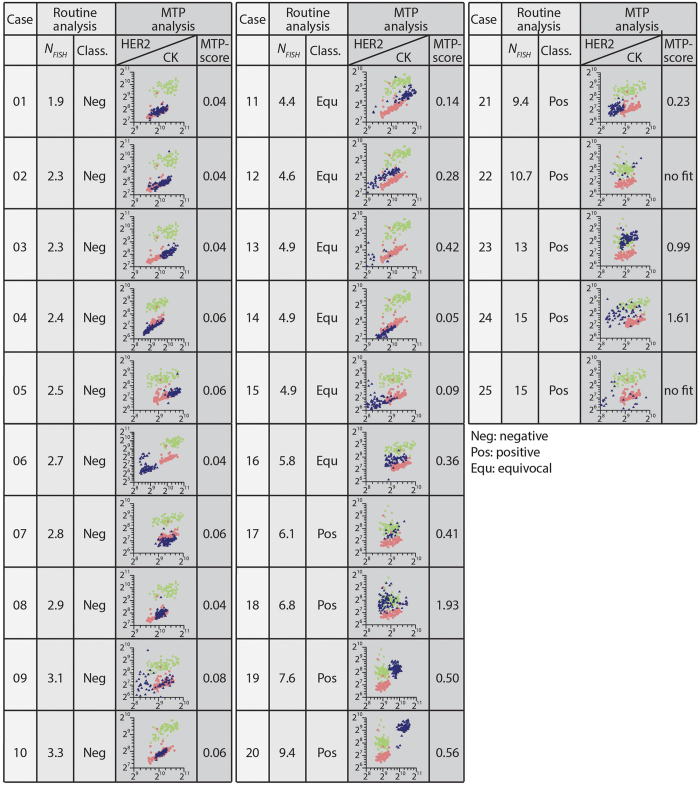
Scatter plot signatures obtained by MTP IF and the corresponding *HER2* gene copy number for 25 invasive breast carcinoma cases. The listed values for the cell-averaged *HER2* gene copy number (*N*_*FISH*_) were obtained from FISH routine analysis. The HER2 status classification (Class.) by the pathologist is also given, based on *N*_*FISH*_ as follows: *N*_*FISH*_ < 4: Negative; 4 ≤ *N*_*FISH*_ < 6: Equivocal; *N*_*FISH*_ ≥ 6: Positive. For the MTP analysis, the 25 cases were grouped into 5 batches and samples were processed sequentially in one run with the MTP, while one positive (IHC 3 + score) and one negative (IHC 0 score) control sample were each time included in the batch. An automated algorithm was used to determine the regions of interest with epithelial cells and remove the background, as illustrated in Fig. S5, for each tile of the mosaic image of a given sample. This resulted in one average HER2 and CK signal per tile, which is represented by a point in a scatter plot for each patient. The scatter plot shows the correlation between the tile-averaged HER2 and CK signals (blue), compared with the scatter plot data obtained from the IHC 3+ (green) and IHC 0 (red) control samples of the batch. Analysis of the scatter plots, as further illustrated in Fig. 3, provided a MTP score for each patient that clearly correlated with *N*_*FISH*_ obtained from routine analysis.

We selected 25 invasive breast carcinoma cases, for which routine FISH analysis gave a wide range of *HER2* gene copy numbers (*N*_*FISH*_) ranging from 1.9 to 15 (see Table S1). IHC results were equivocal in several cases out of this series. Table S1 of the Supplementary Information shows the classification of IHC results for these 25 cases following the 2013 ASCO/CAP interpretation guidelines^5^ by two blinded experienced pathologists (M.F. and B.B.). By routine *HER2* FISH analysis, based on the *N*_*FISH*_ values, 10 cases out of the 25 were classified as negative (*N*_*FISH*_ < 4), 9 cases as positive (*N*_*FISH*_ ≥ 6), and 6 cases as equivocal (4 ≤ *N*_*FISH*_ < 6) (Neg, Pos, and Equ, respectively, in Fig. 2). Although the 2013 ASCO/CAP guidelines for HER2 status classification also takes into account the *HER2*/CEP17 ratio, we decided for the purpose of this study to focus on the correlation between HER2 protein expression and *N*_*FISH*_.

For the MTP analysis, we grouped the 25 cases into 5 batches and processed all samples in a batch sequentially in one experimental run. We used an automated algorithm to define the areas that had epithelial cells and were to be interrogated for the presence of a HER2 signal, from which we obtained the average CK and HER2 signal for each tile of the mosaic image, resulting in the scatter plots of Fig. 2. From a statistical analysis of the latter, we calculated an MTP-score for each sample that clearly correlated with the *N*_*FISH*_ values obtained from routine analysis (see further). At first sight already, the scatter plots of Fig. 2 show that the samples assigned a low *N*_*FISH*_ value (<∼3) have a linear correlation of HER2 with CK, while for *N*_*FISH*_ > ∼5, HER2 values get systematically higher and more dispersed.

### Automated comparison of a scatter plot to those of the control cases can estimate the *HER2* gene copy number

**Figure 3:**
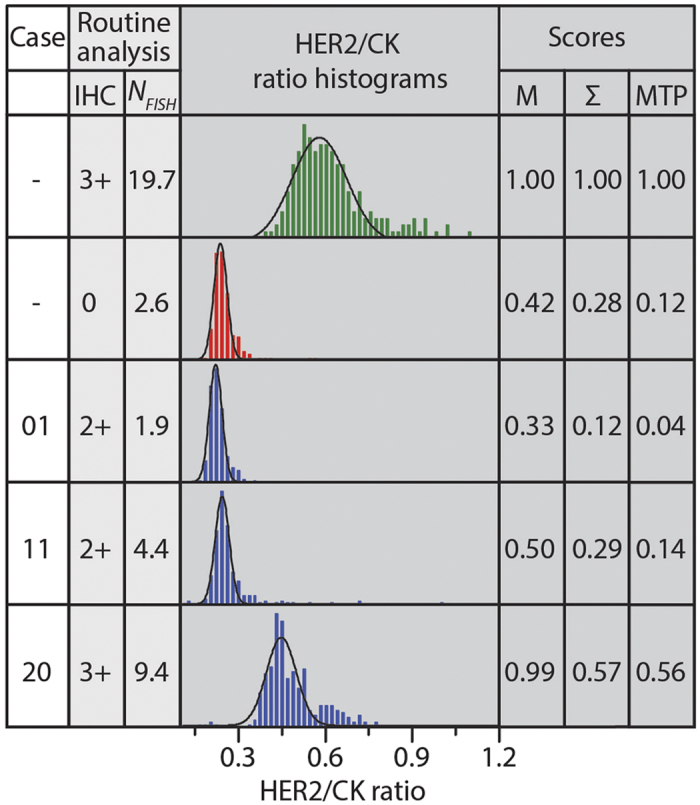
Automated comparison of a scatter plot to those of control cases. Routine analysis prior resulted in following IHC scores: one 3+ case (green), one 0 case (red), and three borderline cases (blue), the latter having *N*_*FISH*_ of 1.9, 4.4, and 9.4, respectively. In our data processing algorithm, the ratio between HER2 and CK signals was obtained on a tile-by-tile basis from the scatter plot data of Fig. 2. The thus obtained population is plotted as a histogram, indicating the frequency of occurrence of a particular ratio; this frequency is normalized by the number of tiles for the given case. A Gaussian fit to the histogram allowed determining the mean HER2/CK value, which was normalized by the mean of the 3+ control sample in the batch, defining the M-score. Similarly, the standard deviation (σ) of the Gaussian fit, normalized by σ of the 3+ control sample, defined the Σ-score. The MTP-score is defined as the product of the M- and Σ-scores.

**Figure 4:**
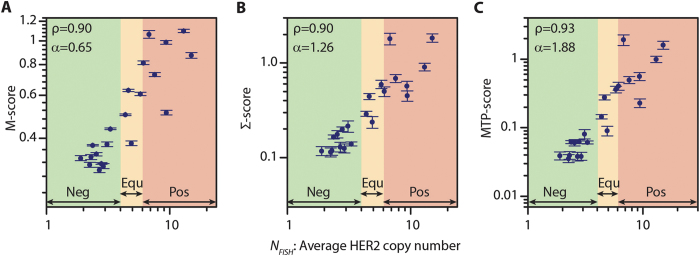
MTP-Score can estimate *N*_*FISH*_ with a high confidence. (**A**) M-score, (**B**) Σ-score and (**C**) MTP-score vs *N*_*FISH*_. The results of the HER2 status classification according to routine FISH analysis are indicated by the green, orange and red zones, representing negative (Neg), equivocal (Equ) and positive (Pos) cases, respectively. ρ and α represent Pearson’s coefficients and slopes of the power law fits, respectively. Error bars are obtained from Gaussian fits to the histogram data.

To exploit the scatter plot data of Fig. 2, we implemented a data processing algorithm that compares the scatter plot of a case under interrogation to those obtained from absolute negative and positive control cases. This algorithm starts by calculating the ratio between HER2 and CK signals on a tile-by-tile basis. The thus obtained data array is then plotted as a histogram, representing the frequency of occurrence of a given HER2/CK ratio normalized to the number of tiles. Figure 3 shows as examples the histograms obtained for five patients, to whom prior routine analysis attributed the following IHC scores: 3+ (green), 0 (red), and three cases (blue) from which two scored as 2+ and one as 3+ and had *N*_*FISH*_ values of 1.9, 4.4, and 9.4, respectively. The positive and negative controls were used as references of the expected HER2 signal intensities for each sample. For the three samples represented in blue, the histograms shifted more towards the right as the *N*_*FISH*_ increased, indicating an average increment in the acquired HER2 signal with respect to CK, when the number of *HER2* gene copies was higher. Moreover, the widening of the histogram for high *N*_*FISH*_ cases showed that the overexpression of HER2 also corresponded to a larger dispersion of the HER2 signal. A Gaussian fit of a histogram allowed determining the mean HER2/CK value and normalizing this by the mean obtained for the IHC 3+ control sample in the batch defined the M-score. Similarly, we extracted the standard deviation (σ) from the Gaussian fit of a histogram and normalized it by the σ value of the positive control of the batch to define the Σ-score. We finally defined an MTP-score for each sample as the product of the M- and Σ-scores. The three scores found by this algorithm showed Pearson correlation coefficients of at least 0.9 against the *N*_*FISH*_ values obtained by routine FISH analysis. Figure 4 shows the score values obtained using our algorithm over the full set of cases used in this study. The M-, Σ-, and MTP-scores obtained a Pearson coefficient ρ of 0.90, 0.90, and 0.93, respectively, and an increasing exponent α of the power law fit (*y ∼ x*^*α*^). This correlation demonstrates that MTP-based IF assays can indeed deliver quantitative information on the overexpression of HER2, which is as precise as the gene copy number obtained by FISH. Similarly, the correlation of the M-, Σ-, and MTP-scores with the *HER2*/CEP17 ratio is depicted in Fig. S1. The correlation in this case is, nevertheless, less performing than in the case of *N*_*FISH*_, giving a Pearson coefficient of 0.84, 0.82 and 0.85, respectively.

Moreover, in an effort to explore the advantages of short incubation times for accurate IHC independent of the image analysis protocol, we have done additional experiments on tissue samples, in which we specifically used an off-chip protocol with long incubation times (typically 1 hour) for the staining, after which IF was assessed using the same automatic image analysis protocol. Figure S2A shows the histograms of the HER2/CK ratio for a few cases, either obtained with (i) the MTP using short incubation times, or (ii) the off-chip protocol using 1 hour incubation. We clearly observe that the histograms become broader and shift to higher HER2/CK ratios for the long incubation time, rendering a less accurate assessment of the HER2 expression level, as evidenced in the plot of the M-score (Fig. S2B) and the Σ-score (Fig. S2C), especially for *N*_*FISH*_ <6, which is the interval for which equivocal results are encountered.

### MTP fine-tunes immunoreactions and produces an IF staining that is linearly proportional to the Ag concentration on the tumor cell surface

**Figure 5:**
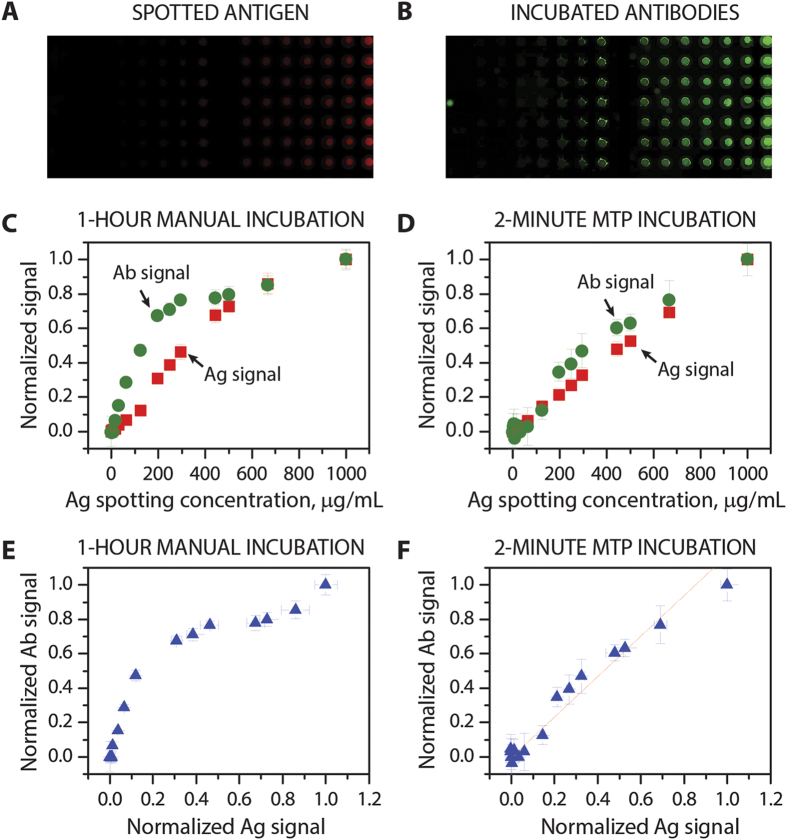
Effect of the Ab incubation time on the proportionality of Ag and Ab signals. (**A**) Fluorescent image of AF647 mouse antibodies used as Ag and spotted on an epoxy-functionalized glass slide with a concentration ranging from 0 to 1000 μg/mL. (**B**) Fluorescent image of AF488 anti-mouse antibodies, used as Ab delivered and incubated using the MTP. (**C**,**D**) show the normalized fluorescent signals of the spotted Ag and its recognizing Ab for an 1 hour and 2 minutes incubation time, respectively, versus the Ag spotting concentrations. (**E**,**F**) depict the normalized fluorescent signals of the recognizing Ab versus the signals of the spotted Ag for an 1 hour and 2 minutes incubation time, respectively. For the short incubation time, the Ab-Ag curve shows a linear-like relation with a regression coefficient of 0.96; this proportionality is in favor of the accuracy of the fast MTP-based staining procedure.

In order to better understand the origins of the precision in the obtained IF staining that can even be used to estimate the gene copy number, we performed a proof-of-concept experiment by immobilizing fluorescently labeled Ags on a glass surface with various volume concentrations, which ranged from 0 to 1000 μg/mL. As depicted in Fig. 5, long incubation times of one hour for manual assays resulted in a non-linear relationship between the Ab fluorescent signal and its Ag, showing sudden Ab signal saturation with respect to its Ag concentration. On the contrary, when the incubation time for the Ab-Ag reaction was limited to 2 minutes and the MTP was used for the staining, we observed a signal from the antibodies that was more proportional to that of the Ags. The linear fit of the Ab-Ag signal plotted in Fig. 5F resulted in a regression coefficient of 0.96. This experiment was performed using fluorescently labeled IgG and showed the advantage of using precise microfluidic IF to significantly reduce the incubation times when compared to current standard protocols that use incubation times from 30 minutes to a few hours. Even though the characteristics of the IgG spotting experiments do not fully correspond to those of the tissue, the recognition process is also based on an Ag-Ab interaction at a surface, like on a tissue slide. The implementation of a spotting microarray allowed us to obtain a direct fluorescence signal from the Ags and compare it to the signal from the recognizing Abs in an analytical fashion. On top of this, this assay allowed us to create a controlled gradient of Ag concentrations on the same slide, which is not possible with tissue sections. Finally, we performed a second incubation with antibodies that recognized the previously incubated IgGs. The results (not shown in this plot) also manifested that a short incubation time of 2 minutes gives a signal that is more proportional to the Ag concentration than an incubation of one hour.

## Discussion

Microfluidic precision IF proved to be very powerful in terms of quantifying Ag expression in histopathological tissue sections, significantly increasing the precision of the information that can be obtained by an immunoassay. Experiments showed that HER2 biomarker quantification, as obtained by the presented method, can provide molecular information that is as precise as data obtained by FISH tests, while keeping the cost and time advantage of an IF assay. We showed that the low incubation times (<8 minutes) allowed by microfluidic staining resulted in a proportional relationship between the IF signal and the corresponding Ag concentration, and that this feature was lost when using higher incubation times, which basically explained the basis of the precision in the results. The linearity of the staining, combined with low-complexity image analysis, allowed us to establish a continuous scoring that linearly followed the gene copy number as assessed by *in situ* hybridization.

Today, ISH is used as a gold standard for *HER2* gene amplification assessment in breast cancer and for other diagnostic applications, and is replaced to some extent by conventional IHC as a surrogate marker of the genetic status. While this first proof-of-concept comparison of the presented method against FISH results is already promising, showing that the same results can be obtained much faster and at a lower cost, the microfluidic precision IF method used to obtain the scatter plots hereby presented has potentially a higher degree of clinical relevance.

FISH assessment is done by averaging the *HER2* gene copy number and the *HER2*/CEP17 ratio of 20 to over 100 nuclei of invasive cancer cells, though intra-tumoral genetic heterogeneity is a well-known phenomenon and a challenge for HER2 evaluation in some breast cancer cases^23^. 2013 ASCO/CAP guidelines have partially clarified this point and stressed the importance of screening the whole section to detect heterogeneous, (partially) amplified cases^5^.

Interestingly, for cases 6, 9 and 14 in this study, heterogeneity was detected by both technical approaches. Routine IHC followed by FISH analysis demonstrated two areas of the tissue expressing/amplifying HER2 at different levels. On the other hand the scatter plots obtained (see Fig. 2) using our method showed that there are 2 distinct populations with different levels of HER2 expression, in comparison to a negative *N*_*FISH*_.

What is more, we do know that gene copy number gain is not always sufficient for protein overexpression. The scatter plot of case 15, having an *N*_*FISH*_ value of 4.9, correlates more with the HER2-negative control signature with MTP-scores less than 0.1, suggesting that although there is a moderate copy number gain, there is no significant protein overexpression. Indeed, case 15 was considered equivocal/positive by FISH analysis, even though the IHC score was +1 (see Table S1).

Further clinical studies comparing the MTP score to clinical outcomes (such as survival and response to anti-HER2 targeted therapies) are required to show if the MTP score can be used to more precisely predict the treatment success in clinical assessment of HER2. In addition to the HER2 biomarker case presented here, another example is anaplastic lymphoma kinase (ALK) biomarker searched in non-small cell lung cancer, where *ALK* gene rearrangementas assessed by FISH is used to define a positive ALK status^24^. However, the response rate to targeted therapy (Crizotinib) remains around 58% for ALK-positive patients diagnosed this way^25^. While ALK protein expression as detected by IHC is progressively being introduced as a surrogate marker for *ALK* gene rearrangement^26^,^27^, one immediate further validation of our approach could involve the quantitative evaluation of ALK expression in non-small cell lung cancers. Finally, the scatter signature and MTP score obtained by precision microfluidic IF can increase the correlation between the biomarker assessment and treatment response, by establishing the missing link between gene status assessment by FISH and protein expression evaluation by IHC.

To conclude, we anticipate that the clinical implication of the presented results is potentially more significant than a mere application of the presented method in eliminating complementary ISH assessment in HER2 expression or other biomarkers. The experiments showing a more linearly proportional IF staining with respect to the Ag expression level, combined with the obtained scatter plots, imply that standard IHC is potentially masking some clinically relevant protein biomarker information. The expansion of this technique and the integration of the obtained information to routine diagnostic workflows may imply a major leap towards the concretization of precision medicine. Microfluidic precision IF uses standard sample preparation techniques and clinically validated primary antibodies. As digital pathology slide scanners are becoming more and more common, a switch to IF scanning would be easier, and does not imply high testing costs as opposed to complementary genetic testing practices like ISH. A low-complexity algorithm, as presented here, can easily generate scatter plots and a score without requiring too much computational power. Such quantitative data can be used as a scoring aid to pathologists, to increase success of treatment response prediction and prognosis. In the future, larger clinical studies for several markers will be required to further validate and prove the impact of microfluidic precision IF technology in cancer diagnosis.

## Methods

### Study design

This pilot study tested the ability to quantitatively score the level of overexpression of the HER2 protein in breast carcinoma samples using the MTP and compare it with the *N*_*FISH*_ value and the *HER2*/CEP17 ratio provided by routine diagnostic. Patient samples were obtained from the Institute of Pathology, Centre Hospitalier Universitaire Vaudois (CHUV), Université de Lausanne (UNIL), Lausanne, Switzerland. Twenty-five samples from patients were carefully selected to span across a wide range of *N*_*FISH*_ values, assessed at the Institute of Pathology according to 2013 ASCO recommendations^5^. For the MTP analysis, the 25 cases were grouped into 5 batches that were processed sequentially. One positive (IHC 3+ score) and one negative (IHC 0 score) control sample was each time included in the batch. The study has been conducted on anonymized tissues of subjects that have not expressed any objection to the use of their tissue, in accordance with the recommendations and after validation by the Ethical Commission of Clinical Research of the state of Vaud (BB511/2012).

### Tissue preparation and reagents

Breast carcinoma samples were obtained from the bio-bank of the Institute of Pathology and provided as 4 μm FFPE sections mounted on Super Frost Plus slides (Menzel-Glaser, Germany). Tissue samples were first dehydrated for 10 min at 65 °C, then dewaxed using Histo-clear (National Diagnostics, GA, USA) for 10 min and rapidly rehydrated using ethanol solutions in decreasing concentrations (100%, 95%, 70% and 40% vol/vol) (Sigma-Aldrich, MO, USA). Subsequently, heat-induced Ag retrieval was done using sodium citrate buffer pH 6 (code: S1699 Dako, Denmark) for 10 min at 95 °C in a hot bath. The samples were then cooled down to room temperature for 20 min and immersed in phosphate buffered saline pH 7.4 (PBS) (Sigma Aldrich, MO, USA). The histological slides were inserted into the device to run the MTP staining assay. PBS was used as a buffer for cleaning and priming of the fluidic path. Double-staining was performed using rabbit anti-human c-erbB-2 oncoprotein (code: A0485, Dako, Denmark) and mouse anti-human cytokeratin, clone AE1/AE3 (code M3515, Dako, Denmark), as primary antibodies, with a concentration of 1.28 μg/mL and 1.02 μg/mL, respectively. For fluorescent detection, Alexa Fluor 594 goat anti-rabbit IgG (H+L) (code: A-11037, Life Technologies, CA, USA) and Alexa Fluor 647 goat anti-mouse IgG (H+L) (code: A-21236, Life Technologies, CA, USA) secondary antibodies, at a concentration of 50 μg/mL, were employed. Nuclear counterstaining was realized using DAPI, included in Fluoroshield (code: F6057, Sigma Aldrich, MO, USA) mounting solution. All the antibodies were dissolved in a 0.05% (vol/vol) solution of Tween 20 (code: P137-9, Sigma Aldrich, MO, USA) in PBS.

### Routine analysis

For routine determination of HER2 status of breast cancer cases at the Institute of Pathology, IHC was performed on 4 μm FFPE sections on the Ventana Benchmark automat (Ventana Medical Systems, AZ, USA). The samples were stained using Ventana anti-HER2/*neu* Ab (clone: 4B5) and scored according to current ASCO/CAP guidelines^5^. FISH was done manually on 4 μm FFPE sections using the PathVysion HER-2 DNA probe kit (Abbott Molecular, IL, USA). Signal analysis was performed on a minimum of 40 nuclei per case after screening of the whole section.

### Device fabrication and experimental setup

The MTP was designed using Virtuoso (Cadence, CA, USA) and then fabricated via deep reactive ion etching of microfluidic channels on a silicon substrate, followed by bonding of a Parylene C-coated Pyrex wafer (Fig. S3 of the Supplementary Information), thereby forming 100-μm high and 250-μm wide microfluidic channels. The details of this fabrication technique can be found elsewhere^15^,^28^,^29^. The MTP formed a fluidic halve-chamber that was reversibly clamped with a tissue slide using the force provided by a permanent magnet (Fig. S4). When forming the MTP-glass slide fluidic chamber, the histological glass slide was clamped against the MTP via a polydimethylsiloxane gasket, both to fix the height of the reaction chamber to 100 μm and to prevent leakage. For interfacing the MTP with external fluidic control systems, a polymethylmetacrylate holder is assembled with the MTP. Fluid manipulation was realized using five syringe pumps (Cetoni, Germany) that were filled with the required reagents and connected to the inlet of the MTP via the holder (Fig. S4).

### MTP IF staining protocol

After clamping the MTP with the glass slide, the staining protocol lasted 10 minutes and is detailed in Table S2. PBS solution, used to wash the chamber in between steps, was delivered at 25 μL/s for 10 seconds. Ab solutions were delivered at 10 μL/s for 12 seconds, and incubated for 2 minutes with a slow flow of 20 nL/s. Upon finalization of the staining protocol, the tissue samples were washed off-chip with deionized water for ten seconds and mounted using 170-μm coverslip using a DAPI-containing solution. For our experimental design related to the incubation time, we considered the typical values of IgG Ag-Ab binding constants k_on_ (~10^6^ M^−1^) and k_off_ (~10^−3^  s^−1^)^30^,^31^. The bulk Ab concentration c_bulk_ (~10^−8^ M) was chosen large enough so that it is not a limiting factor for the Ab surface coverage and the IF signal in a Langmuir isotherm hypothesis. The binding constants allow to calculate the desorption time t_d_ = 1/k_off_ ~ 10^3^ s, while the time constant of the recognition reaction is τ = 1/(k_on_ c_bulk_ + k_off_) ~ 10^2^  s. This forms the basis for our choice of a few minute incubation times.

### Fluorescence image acquisition

Slides were inserted in an automated epi-fluorescent microscope (Axio Imager M2m, Zeiss, Germany) and mosaic images were obtained using a CCD camera. Images in three fluorescent channels, corresponding to the signals of DAPI, CK and HER2, respectively, were automatically obtained. Autofocusing, acquisition, scanning and stitching were done automatically. Prior to analysis, all images were checked if they contained artifacts that could influence the analysis. Based on this assessment, cases 22 and 25 were removed from the dataset due to the lack of epithelial cells in the stained slide that eventually resulted in a Gaussian fit with a low adjusted R^2^ value of 0.4837 and 0.4806, respectively. This was already evident from the scatter plot in Fig. 2 that indicated a lack of tiles for that case. The average image acquisition varies from 10 to 40 minutes, depending on the size of the sample.

### Determination of the regions of interest for interrogation of the HER2 signal

HER2 protein is situated on epithelial cell membranes. We performed double-staining IF assays using CK as a second marker in addition to the HER2 analysis, due to its expression in all epithelial cells, so that the information on the CK channel could be used to define the areas where the expression of HER2 should be interrogated. ImageJ macros were applied tile-by-tile (n = 40482). To do this, we built an auto-thresholding algorithm that determines where CK is expressed to define the region of interest. Similarly, the information in the DAPI channel was used to identify the nuclei as areas that should not be interrogated, as HER2 does not constitute a nuclear marker. In HER2-overexpressing samples (Fig. S5A), the use of HER2 signal alone would be clearly sufficient to determine the regions of interest; for tumors that do not strongly overexpress HER2 (Fig. S5B), it would be difficult to distinguish epithelial cells from surrounding parts in the tissue. Especially in these cases, employing the signal in the CK channel allowed us to assure that the regions of interest for interrogation of the HER2 signal corresponded to epithelial cells, independent from the intensity of the HER2 signal.

As a result of this procedure, every tile was assigned an average signal value both for CK and HER2. The thus obtained CK signal intensities were analyzed and filtered, in a case-by-case fashion, in order to remove from further analysis the tiles that had none or a few epithelial cells. In particular, these tiles showed a CK average that felt below a given threshold and were automatically filtered out from the dataset (Fig. S6). Finally, *de facto* establishing an upper threshold of the CK signal, we removed the 5% brightest tiles for each case, to account for possible artifacts like small agglomerates of fluorophores that eventually result in saturation of the fluorescent signal intensity. All tiles that showed a CK value above the lower and below the upper thresholds were kept for further analysis. The detailed steps of the image-processing algorithm can be found in Table S3 of the Supplementary Information. OriginLab software (OriginLab Corporation, MA, USA) was used to obtain scatter plots, histograms and statistical values. The image-processing algorithm takes approximately 20 minutes per batch, giving an average of 3 minutes per sample.

### Spotting and incubation of fluorescently labeled antibodies on functionalized slides

Fluorescently labeled AF647 mouse antibodies (code: A-21239, Life Technologies, CA, USA) were spotted on epoxy-functionalized glass slide (Super Epoxy slides, Arrayit, CA, USA) with a concentration ranging from 0 to 1000 μg/mL using a commercially available contact spotter (QArray Mini, Genetix, MA, USA). The MTP-based incubation of recognizing antibodies was done using AF488 anti-mouse antibodies (code: A-11034, Life Technologies, CA, USA), with a concentration of 50 μg/mL. ImageJ macros were used to segment the spot areas and obtain the average fluorescent signal. OriginLab software (OriginLab Corporation, MA, USA) was used to plots the results.

## Additional Information

**How to cite this article**: Dupouy, D. G. *et al*. Continuous quantification of HER2 expression by microfluidic precision immunofluorescence estimates *HER2* gene amplification in breast cancer. *Sci. Rep*. **6**, 20277; doi: 10.1038/srep20277 (2016).

## Supplementary Material

Supplementary Information
