# Supplementary material for: Continuous quantification of HER2 expression by microfluidic precision immunofluorescence estimates HER2 gene amplification in breast cancer
Source: Sci Rep. 2016 Feb 9;6:20277. doi: 10.1038/srep20277 (PMC4746572; doi:10.1038/srep20277)
Supplement: Supplementary Information [file srep20277-s1.pdf]

Supplementary Information

Continuous quantification of HER2 expression by microfluidic precision immunofluorescence estimates *HER2* gene amplification in breast cancer

Authors: Diego G. Dupouy[1], Ata Tuna Ciftlik[1,3], Maryse Fiche[2], Déborah Heintze[1,3], Bettina Bisig[2], Laurence de Leval[2] and Martin A. M. Gijs[1,]*

Affiliations:

[1]Laboratory of Microsystems, Ecole Polytechnique Fédérale de Lausanne, CH-1015, Switzerland.

[2]Institute of Pathology, Centre Hospitalier Universitaire Vaudois and University of Lausanne, CH-1011 Lausanne, Switzerland.

[3]Lunaphore Technologies SA, EPFL Innovation Park - Building C, CH-1015, Lausanne, Switzerland

*Corresponding author. e-mail: martin.gijs@epfl.ch (M.A.M.G.)

Supplementary notes:

Table S1. Results of the routine IHC and FISH analyses for all cases.

Fig. S1. Score vs. HER2/CEP17 ratio for all patients

Fig. S2. Comparison of the use of short vs long Ab incubation times for accurate IF analysis.

Fig. S3. Fabrication of the MTP

Fig. S4. Experimental setup

Table S2. MTP immunohistochemical staining protocol

Fig. S5. Example of image tile processing for MTP analysis

Fig. S6. Use of CK filter to obtain the HER2 histogram

Table S3. Sequence of the image and data processing algorithm

Table S1. Results of the routine IHC and FISH analyses for all cases. Comparison is made between: i) *IHC scoring* following 2013 ASCO/CAP guidelines *(7)*; ii) N_{FISH} ; iii) *HER2/CEP17 ratio*; iv) *HER2 status classification based on* N_{FISH} (N_{FISH} <4: Negative; 4≤ N_{FISH} <6: Equivocal; N_{FISH} ≥6: Positive) and v) *HER2 status classification* according to 2013 ASCO/CAP guidelines *(7)*. The aim of this process was to compare MTP results to the results of traditional IHC at the time of the study. Case 13 was removed from the dataset in Fig. 4 since a control HE slide showed that only ductal carcinoma *in situ* (DCIS) was left on the sections used for the study. Similarly, case 14 was removed from the dataset in Fig. 4 because the section presented heterogeneous HER2 status, resulting in two N_{FISH} values. For cases 22 and 25, interpretation of routine IHC on the resected tumor specimen failed due to repeated tissue detachment from the glass slide. The IHC score indicated for these cases is the one found on the initial core biopsy.

Case	i) IHC scoring	ii) N_{FISH}	*iii) HER2/*CEP17 *ratio*	*iv)* HER2 status based on N_{FISH}	v) HER2 status classification
1	2+	1.9	0.76	negative	negative
2	2+	2.25	1.06	negative	negative
3	1+	2.30	1.01	negative	negative
4	1+	2.38	1.29	negative	negative
5	1+	2.54	1.20	negative	negative
6	0	2.70	1.19	negative	negative
7	0	2.80	1.22	negative	negative
8	2+	2.90	1.12	negative	negative
9	2+	3.10	1.60	negative	negative
10	1+	3.30	1.38	negative	negative
11	2+	4.38	1.80	equivocal	equivocal
12	3+	4.64	1.48	equivocal	positive
13	2+	4.85	2.09	equivocal	positive

	(only DCIS left)				
14	2+	4.90/2.50	2.7/1.3	equivocal (heterogeneous)	positive (heterogeneous)
15	1+	4.90	2.30	equivocal	positive
16	1+	5.80	2.80	equivocal	positive
17	2+	6.13	2.08	positive	positive
18	2+	6.80	1.80	positive	positive
19	3+	7.60	3.30	positive	positive
20	3+	9.35	5.30	positive	positive
21	3+	9.40	2.60	positive	positive
22	3+	10.70	4.90	positive	positive
23	3+	13.00	3.40	positive	positive
24	3+	15.00	3.00	positive	positive
25	3+ (on the core biopsy)	15.00	7.50	positive	positive

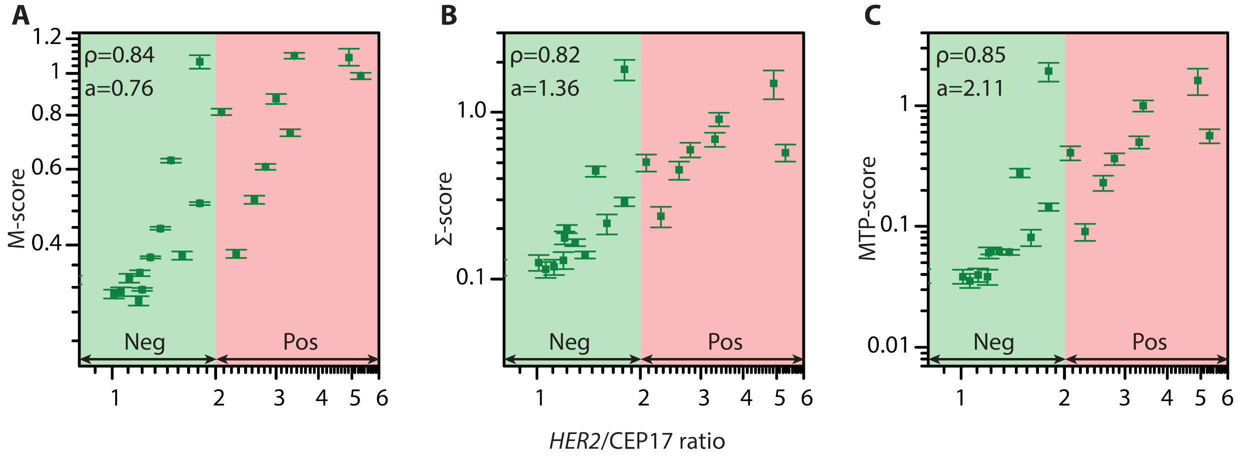

Fig. S1. **Score vs. *HER2*/CEP17 ratio for all patients.** (**A**) M-score, (**B**) Σ-score and (**C**) MTP-score vs *HER2*/CEP17. The results of the HER2 status classification according to routine FISH analysis are indicated by the green and red zones, representing negative (Neg) and positive (Pos)

cases, respectively. ρ and α represent Pearson's coefficients and slopes of the power law fits, respectively. Error bars are obtained from Gaussian fits to the histogram data.

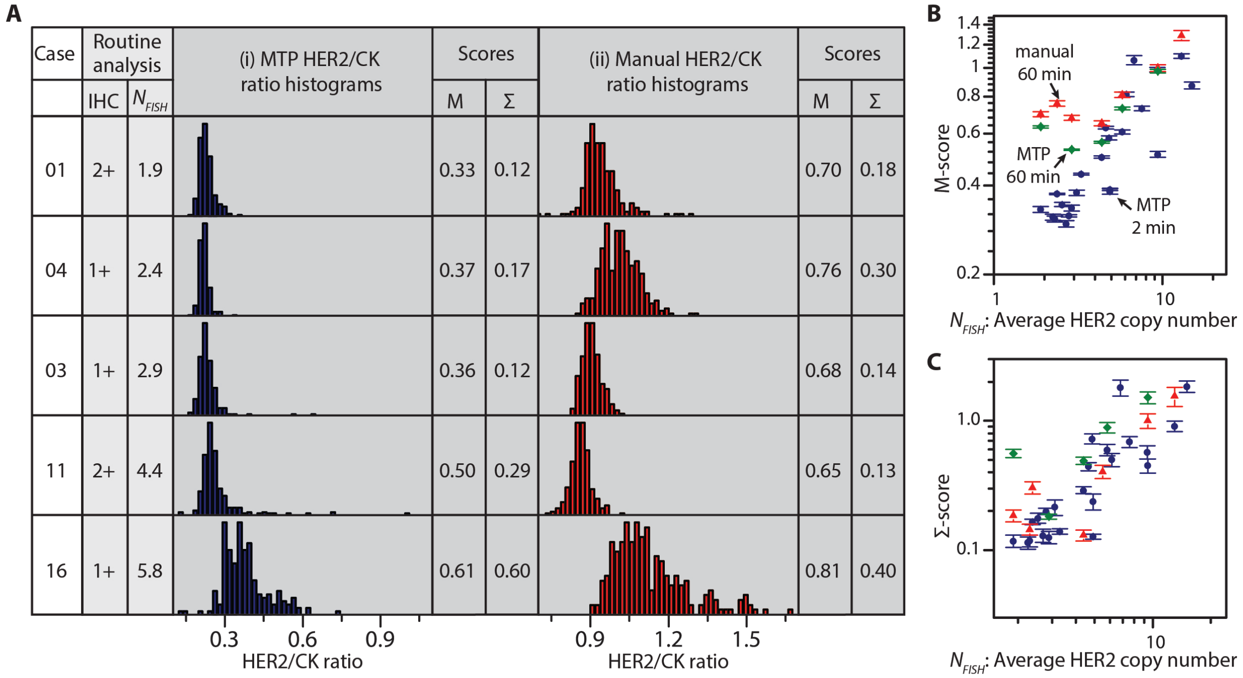

Fig. S2 Comparison of the use of short vs long Ab incubation times for accurate IF analysis. (A) Histograms of the HER2/CK ratio for a few cases, either obtained with (i) the MTP using short incubation times, or (ii) an off-chip protocol using 1 hour incubation. We clearly observe that the histograms become broader and shift to higher HER2/CK ratios for the long incubation time. The broadening leads to a less accurate assessment of the HER2 expression level, as evidenced in the plot of the M-score (B) and the Σ-score (C), especially for $N_{FISH} < 6$, which is the interval for which equivocal results are encountered. The red points correspond to experiments done with the 1 hour incubation time, while blue points were obtained with the MTP using a 2 min incubation time protocol. The green points represent additional control stains done with the MTP using a long incubation time of 1 hour. These results show that the obtained automated scores when using long incubation times have little or no diagnostic value for low N_{FISH}, the interval where quantitative results are most required in practical diagnostics. Using short incubation times with MTP not only solves this problem but also provides a much more proportional score to quantitative results obtained by in-situ hybridization in all diagnostic range.

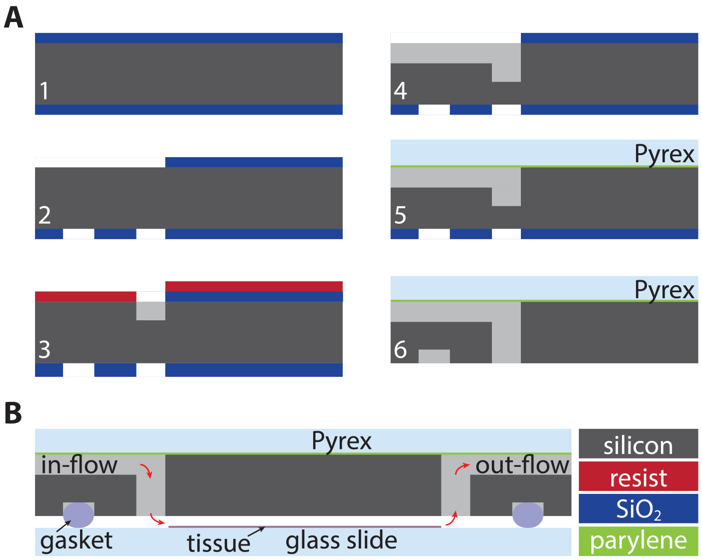

Fig. S3. **Fabrication of the MTP.** (**A**) Clean room fabrication steps: (1) silicon wafers with a 2-μm layer of wet silicon oxide; (2) micropatterning of the oxide into a hard mask using deep reactive ion etching (DRIE); (3) micropatterning of silicon by DRIE via a photolithographic step to structure vertical holes; (4) resist stripping and subsequent DRIE of silicon via the hard mask to structure the microfluidic channels, followed by removal of the hard mask; (5) bonding of the silicon wafer to a parylene C-coated Pyrex wafer; (6) back side opening of the holes and the gasket notch by DRIE and final removal of the hard mask. (**B**) A schematic cross-section of the finalized MTP, assembled with a tissue slide via a PDMS gasket.

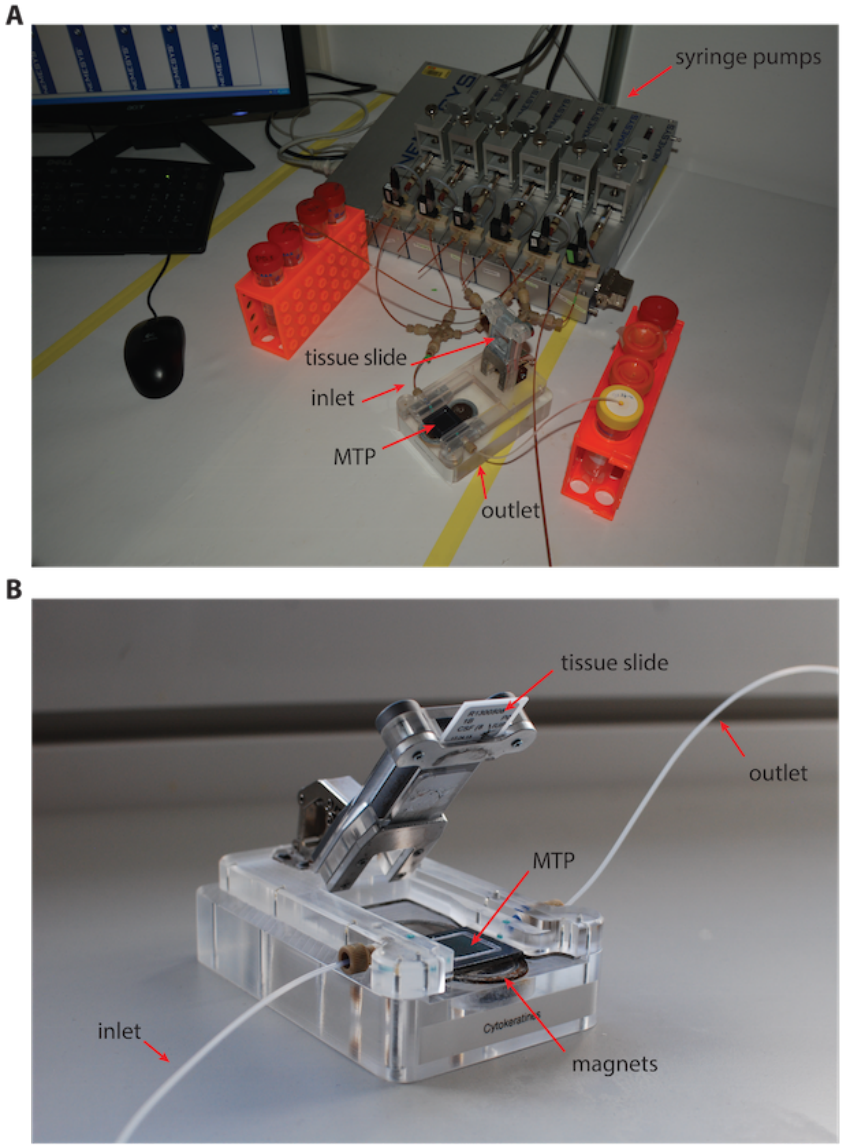

Fig. S4. **Experimental setup.** (**A**) Overview of the experimental setup. **(B)** Assembled MTP; the microfluidic chamber, formed by clamping the tissue slide to the MTP, is opened to show the different parts and the loading mechanism.

Table S2. MTP immunohistochemical staining protocol

Reagent	Flow duration, s	Incubation time, min	Total time, s
PBS buffer	10	-	10
Anti-human cytokeratin, clone AE1/AE3	12	2	132
PBS buffer	10	-	10
Anti-human c-erbB-2 oncoprotein	12	2	132
PBS buffer	10	-	10
AF 594 goat anti-rabbit IgG (H+L)	12	2	132
PBS buffer	10	-	10
AF 647 goat anti-mouse IgG (H+L)	12	2	132
PBS buffer	10	-	10
**Total**	**98**	**8**	**578**

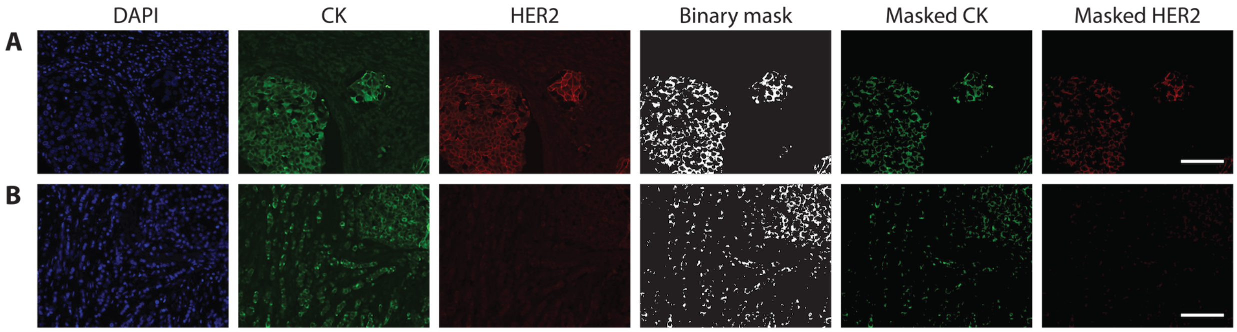

Fig. S5. Example of image tile processing for MTP analysis. The image processing algorithm is applied to a case where HER2 is overexpressed (**A**) and not expressed (**B**). The algorithm uses the DAPI channel to mask the locations of the nuclei, as they constitute an area that should not be examined, while the CK channel indicates the location of epithelial cells, i.e. exactly the areas where the HER2 and CK signals should be interrogated. This allows constructing a binary mask

by which one can assign a single tile-averaged value for the CK and HER2 signals, obtained from the areas of interest only. Scale bar: 100 µm.

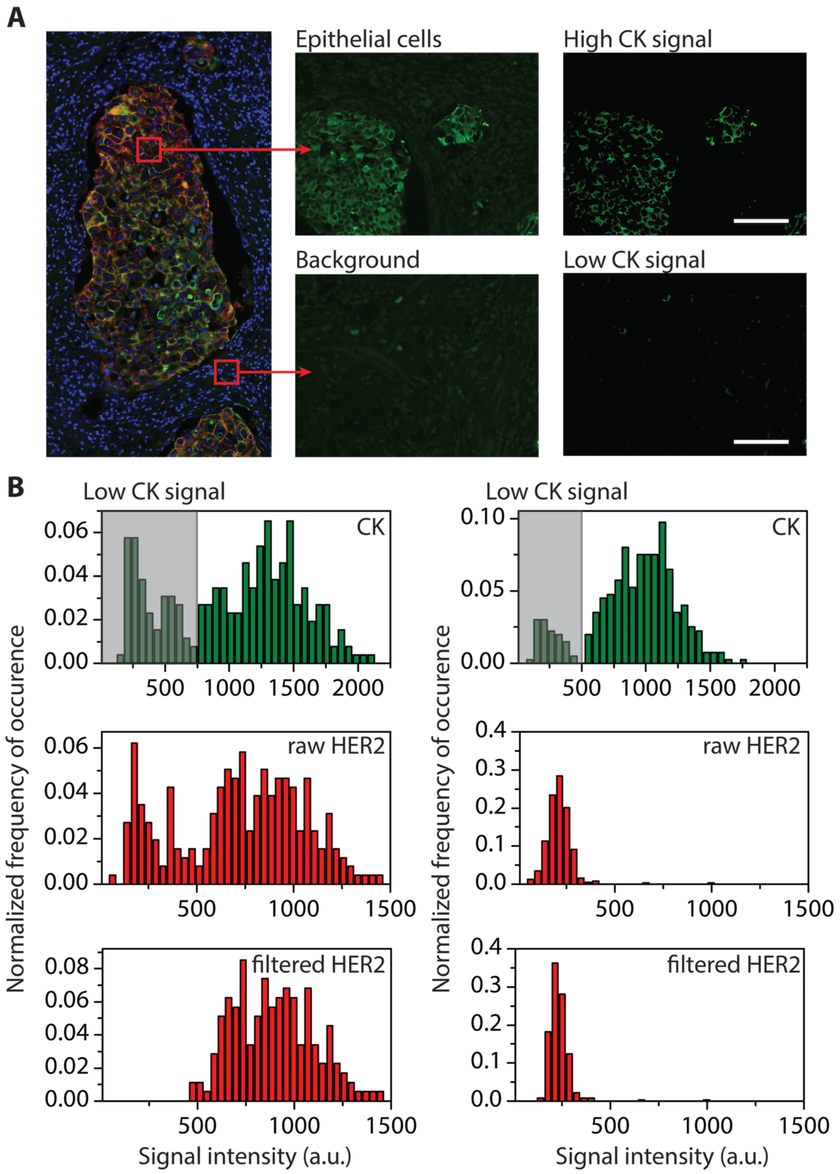

Fig. S6. Use of CK filter to obtain the HER2 histogram. (**A**) Example of tissue areas with (top) and without (bottom) epithelial cells and visualized in the raw CK channel (two middle images) and after running the image processing algorithm (two right images). (**B**) Raw histograms of the intensity distribution of the CK (green, first row) and HER2 (red, second row)

signals for a HER2 IHC 3+ case (left column) and a HER2 IHC 0 case (right column). The tiles that show a low CK signal intensity are removed from the histogram and the third row shows the HER2 histograms plotted after application of this CK filt er. The latter histograms represent the data sets for further analysis.

Table S3. **Sequence of the image and data processing algorithm.**

inputs		operation	output
Tile-by-tile processing			
Raw CK tile	-	autothresholding	CK mask
Raw DAPI tile	-	autothresholding	DAPI mask
CK mask	DAPI mask	Binary product	Epithelial cells mask
Raw CK tile	Epithelial cells mask	Signal extraction of relevant regions only	Average CK signal
Raw HER tile	Epithelial cells mask	Signal extraction of relevant regions only	Average HER2 signal
Average CK signal	-	FOR loop (all tiles)	CK data matrix
Average HER2 signal	-	FOR loop (all tiles)	HER2 data matrix
Data matrix processing			
CK data matrix	-	autothresholding	Filtered CK matrix
HER2 data matrix	-	autothresholding	Filtered HER2 matrix
Data analysis			
Filtered CK matrix	-	binning	CK histogram
Filtered HER2 matrix	-	binning	HER2 histogram
CK histogram	HER2 histogram	x-y plot	HER2-CK scatter plot
CK histogram	HER2 histogram	Ratio calculation	HER2-CK ratio histogram
HER2-CK histogram		Gaussian fit	Mean and sigma
